# Molecular Regulation Mechanism of Microglial Autophagy in the Pathology of Alzheimer's Disease

**DOI:** 10.14336/AD.2023.0106

**Published:** 2023-08-01

**Authors:** Pei Ou-Yang, Zhi-Yu Cai, Zhong-Hao Zhang

**Affiliations:** ^1^Shenzhen Key Laboratory of Marine Bioresources and Ecology, College of Life Sciences and Oceanography, Shenzhen University, Shenzhen, China.; ^2^Shenzhen Bay Laboratory, Shenzhen, China.; ^3^Shenzhen-Hong Kong Institute of Brain Science-Shenzhen Fundamental Research Institutions, Shenzhen, China

**Keywords:** Alzheimer's disease, autophagy, microglia, β-amyloid;, receptors

## Abstract

Alzheimer’s disease (AD) is a neurodegenerative disorder characterized by the progressive accumulation of abnormal protein aggregates, neuronal loss, synaptic dysfunction, and neuroinflammation. Microglia are resident macrophages of the central nervous system (CNS). Evidence has shown that impaired microglial autophagy exerts considerable detrimental impact on the CNS, thus contributing to AD pathogenesis. This review highlights the association between microglial autophagy and AD pathology, with a focus on the inflammatory response, defective clearance, and propagation of Aβ and Tau, and synaptic dysfunction. Mechanistically, several lines of research support the roles of microglial receptors in autophagy regulation during AD. In light of accumulating evidence, a strategy for inducing microglial autophagy has great potential in AD drug development.

Alzheimer's disease (AD) is a neurodegenerative disease of the central nervous system (CNS), characterized by β-amyloid (Aβ) protein deposition, abnormal phosphorylation of the Tau protein, synaptic dysfunction, and neuroinflammation [1]. AD is also considered a chronic neuroinflammatory disease, as inflammation plays a critical role in its pathogenesis. An early study reported that various inflammation markers, including immunoglobulins, complement factors, and acute-phase proteins, were enriched in the senile plaques of AD patients [2]. In addition, proinflammatory cytokines (interleukin 1β (IL-1β), interleukin 6 (IL-6), and tumor necrosis factor α [TNF-α]) are markedly increased in patients with mild cognitive impairment and AD [3-6], suggesting that intrathecal inflammation precedes AD development.

Microglia are the predominant immune cell type within the CNS, with significant roles in a number of pathological conditions. Studies have established that abnormal microglia activation is linked to neuroinflammation, a risk factor for AD [7]. Recently, researchers uncovered a novel microglia type, disease-associated microglia (DAM)/microglial neurodegenerative phenotype, in an AD mouse model harboring five human familial AD gene mutations (5×FAD). These AD-associated microglia were observed in 5×FAD mice and postmortem AD patient brains but were scarce in the brains of wild-type mice [8]. Gene Ontology analysis of DAM-specific genes previously revealed significant involvement in lysosomal/phagocytic pathways and endocytosis [8], implicating the enhanced phagocytic activity of DAMs as the characteristic of a potentially protective microglia subtype produced to phagocytize misfolded or aggregated proteins.

Autophagy is a self-degradative process that removes misfolded or aggregated proteins, clears damaged organelles, eliminates pathogens, and maintains intracellular homeostasis [9]. It is mediated by a set of autophagy-related genes (Atg), with there being an established relationship between mutations in *Atg* and human diseases, particularly neurodegenerative disorders, inflammatory conditions, and cancers [10]. Cho *et al*. [11] reported that impaired Aβ degradation was observed in microglia-specific *Atg7* knockout mice and si-*Atg7*-transfected microglia cells, supporting the role of autophagy in microglial Aβ degradation. In addition, microglial autophagy is implicated in Tau propagation, with defective microglial autophagy caused by the deletion of *Atg7* aggravating intraneuronal Tau pathology and spread [12]. These lines of evidence suggest that microglial autophagy could regulate AD pathogenesis. However, the specific roles of microglial autophagy in this process have not yet been systematically summarized.

Several signal transduction pathways involved in autophagy have been identified over the past decade, with mammalian target of rapamycin (mTOR) complex 1 (mTORC1) acting as the master negative regulator of autophagy [13]. Adenosine monophosphate-activated protein kinase (AMPK) mediates autophagy induction under various cellular stress conditions. Both AMPK and mTOR regulate autophagy through the direct phosphorylation of mammalian autophagy-initiating kinase ULK1 [14]. In addition, the p38 mitogen-activated protein kinase (MAPK)-dependent phosphorylation of ULK1 mediates autophagy and inflammatory responses in microglial cells [15]. Recent studies have indicated that a group of AD-associated microglial receptors regulate autophagic signaling pathways in AD pathogenesis, as observed for the binding of microglia-expressing receptor (triggering receptor expressed on myeloid cells 2 (TREM2)) with DNAX-activating protein 12 (DAP12), which triggers PI3K activation. Downstream, Protein kinase B (PKB, also called Akt), activates mTORC1 and inhibits autophagy [16]. Although multiple microglia-expressed receptors have been associated with autophagy, little is known regarding their exact regulatory mechanisms in the context of AD progression.

**Figure 1.**
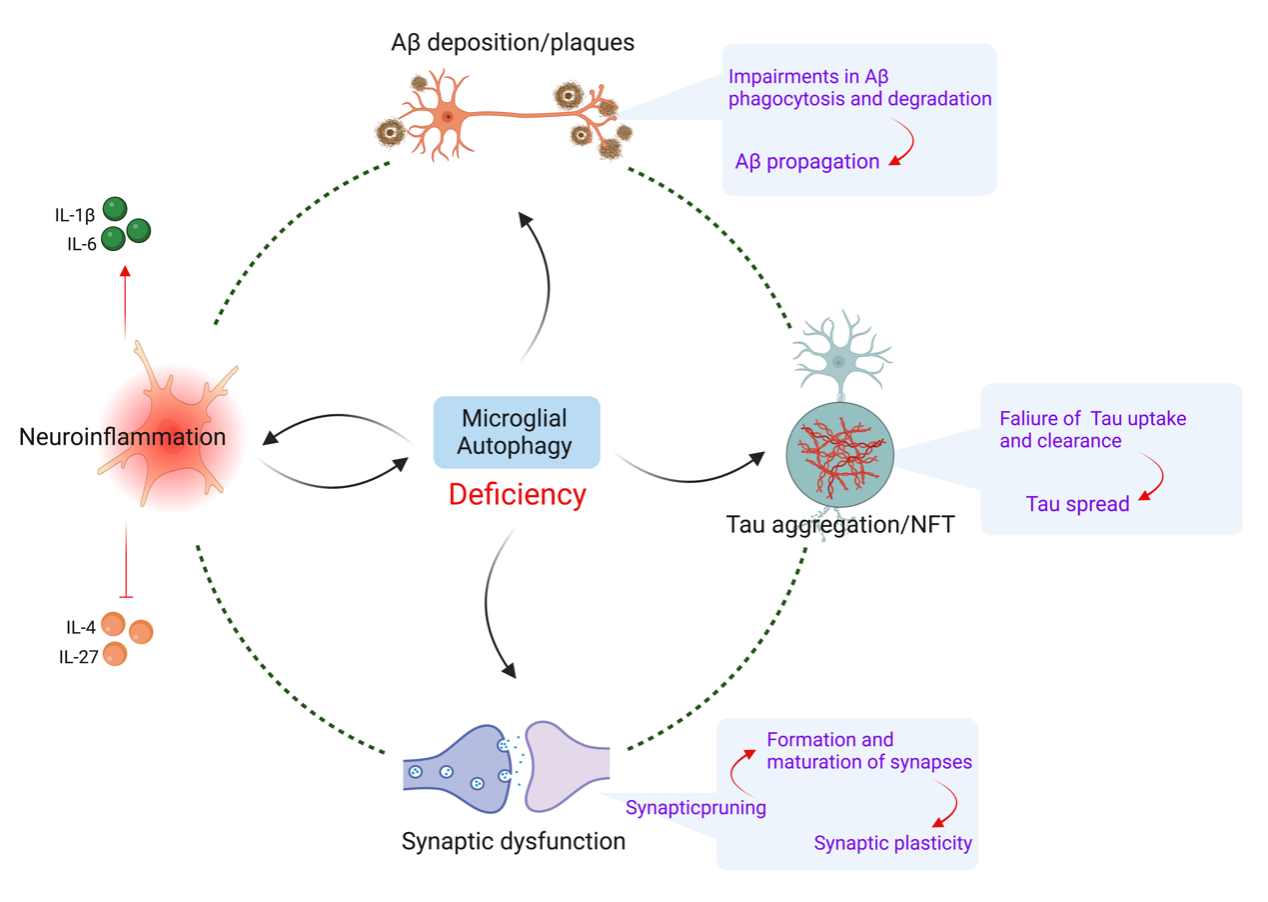
**Relations between microglial autophagy and AD pathogenesis**. Impaired microglial autophagy contributes to multiple aspects of AD pathogenesis including neuroinflammation, Aβ deposition/plaques, Tau aggregation/NFT, and synaptic dysfunction. An interplay exists between microglial autophagy and neuroinflammation in AD. Defective microglial autophagy leads to the accumulation of pathological AD-associated protein (Aβ and Tau) aggregates as a result of impaired Aβ phagocytosis and degradation, Aβ propagation, failure of Tau uptake and clearance, and Tau spread. Finally, microglial autophagy plays a crucial role in modulating synaptic pruning and synaptic plasticity in AD.

This review summarizes the relationship between microglial autophagy and AD pathology, with a focus on neuroinflammation, abnormal protein accumulation, and synaptic dysfunction. The roles of microglial receptors (in autophagy regulation) and the significance of microglial autophagy in AD pathogenesis are discussed. Finally, future perspectives for microglial autophagy-associated strategies in AD treatment are discussed.

## Microglial autophagy and AD pathogenesis

The etiology of AD is related to the abnormal aggregation of pathological proteins. DAMs in the brain of AD patients play a critical role in the phagocytosis and clearance of such pathological proteins. Recent evidence suggests that autophagy is defective in AD, with impaired autophagic clearance contributing to AD pathogenesis. Therefore, understanding the inter-regulation between microglial autophagy and AD pathogenesis is necessary to establish the molecular pathophysiology of AD (Fig. 1).

### Neuroinflammation

Numerous studies have established the interplay between microglial autophagy and neuroinflammation in AD. However, the induction of autophagy negatively regulates inflammasome activation and inflammatory cytokine production by microglia. Microglia-specific deletion of *Atg7* promotes the transition of microglia toward a proinflammatory phenotype and upregulates pro-inflammatory cytokine levels *in vivo*. *Atg7* gene knockout in BV2 cells induces a proinflammatory response and inflammasome activation [12]. Houtman *et al*. [17] reported that increased expression levels of IL-1β and IL-18 are found in acutely stimulated microglia from Becn1^+/-^ mice with impaired autophagy. Notably, a close association of NLRP3 aggregates and LC3-positive vesicles is observed in *Becn1*^+/-^ microglia, implying that defective autophagy modulates microglial IL-1β and IL-18 production via NLRP3. Considering the strongly reduced protein levels of BECN1/beclin-1 in microglia isolated from AD patients [18], these data support a role for impaired autophagy in driving AD-associated neuroinflammation.

Inflammasomes regulate autophagy, mediating the clearance of harmful substrates or secondary signals. NALP3 inflammasome activation triggers caspase-1 cleavage, negatively regulating autophagy via TLR4-TRIF microglial signaling [19]. Knockdown of lincRNA-Cox2 (a newly identified inflammasome modulator) upregulates microglial *Atg5*-dependent autophagy by inhibiting NLRP3 expression [20]. In addition, some cytokines have been documented to regulate autophagy and accelerate neurodegenerative disease progression. In a mouse model of AD, intraperitoneal injection of IFN-γ restored microglial autophagy, promoted amyloid-β clearance, and rescued cognitive deficits. Moreover, IFN-γ protected BV2 cells from Aβ toxicity by upregulating autophagic flux, likely via inhibition of the Akt/mTOR pathway [21]. IL-4, cytokine with protective roles in AD, has been demonstrated to induce the formation of autophagic vacuoles and promote autophagic flux in microglia. Notably, in Aβ-induced microglia, IL-4 pretreatment restored the decreased autophagic flux back to normal, thereby increasing Aβ uptake and degradation [22]. IL-27 exhibits neuroprotective activity and is essential for maintaining CNS integrity. The role of IL-27 in autophagy regulation has been documented in different studies. For example, IL-27 promoted the survival of *Mycobacterium tuberculosis*-infected macrophages by inhibiting autophagy [23]. Meanwhile, Laverdure *et al*. reported that IL-27 promotes autophagy in stimulated primary human macrophages [24]. These contrasting effects of IL-27 reported in previous studies warrant further investigation into the matter. Other cytokines, such as TNF-α [25], IL-1β [26], IL-6 [27, 28], IL-10 [29], IL-17A [30], and IL-33 [31], also regulate autophagy. However, the relationship between the microglial autophagy regulation and the involvement of these cytokines in AD remains unclear.

Generally, microglial autophagy negatively regulates neuroinflammation, thus preserving brain homeostasis. However, excessive activation of the inflammatory response impairs microglial autophagy, further aggravating neuroinflammation in AD. Taken together, the available evidence reveals a close link between microglial autophagy and inflammation in the context of AD pathogenesis. An improved understanding of defective microglial autophagy and how it relates to neuroinflammation in AD is therefore crucial for developing novel therapeutic strategies for AD.

### Pathological proteins in AD

The failure of Aβ and Tau clearance in the CNS is established as a contributing factor to their accumulation in AD brains. Various mechanisms of brain Aβ and Tau clearance have been postulated, ascribing a significant role to microglia in the process. Microglia accumulate around senile plaques in the brains of AD patients, directly phagocytosing Aβ plaques and thereby restricting amyloid plaque formation [32-34]. In addition, lines of evidence suggest the involvement of microglial autophagy in Aβ clearance. Wani *et al*. [35] reported that autophagy activation significantly increases Aβ clearance in microglia, while reducing neuroinflammation and improving memory in the 5×FAD mouse model of AD. Another study reported that extracellular Aβ fibrils (fAβ) can be internalized by microglia to then induce microglial autophagy. Interestingly, internalized fAβ interact with MAP1LC3B-II via OPTN/optineurin and are subsequently degraded via microglial autophagic processes involving the serine/threonine kinase 11 protein kinase, AMP-activated, α-1 catalytic subunit pathway [11]. Luo *et al.* [34] demonstrated that pharmacological activation of peroxisome proliferator-activated receptor alpha (PPARA) induces autophagy in human microglia cells and in the hippocampal and cortex tissues of APP-PSEN1ΔE9 mice. PPARA agonists were shown to reduce amyloid pathology in APP-PSEN1ΔE9 mice, as indicated by lower levels of soluble Aβ and lesser amyloid plaque deposition.

Several studies have demonstrated that microglial autophagy enhances phagocytosis to promote Aβ clearance, thereby attenuating AD progression. Reduced beclin-1 in BV2 microglial cells impairs the phagocytosis of Aβ [18]. The defective function of different macrophage receptors is involved in phagocytosis regulation. The genetic reduction of beclin-1-driven phagocytosis is associated with phagocytic receptor TREM2 recycling [18]. A recent study demonstrated that autophagy regulated macrophage phagocytosis by modulating the expression of scavenger receptors, such as macrophage receptor with collagenous structure (MARCO) and macrophage scavenger receptor 1 [36]. These findings provide insights for further study on the regulation of microglial autophagy during Aβ clearance.

It is generally accepted that Aβ prion-like seeding represents the early stage of plaque formation. Recent studies in AD mouse models implicate activated and phagocytic microglia in Aβ seeding [37-39]. Errico *et al*. [40] highlighted the contribution of microglia to the propagation of Aβ pathology into unaffected brain tissue. Besides, an *in vivo* study using APP transgenic mice showed that autophagy deficiency caused by the conditional knockout of *Atg7* drastically reduces extracellular Aβ plaque burden by influencing Aβ secretion into the extracellular space, which suggests a critical role for autophagy in Aβ secretion and plaque formation [41]. However, there is no direct evidence on the matter. As described above, defective autophagy can induce inflammasome activation. In AD, Aβ deposition is commonly accompanied by activation of the innate immune system and inflammasome. Under these conditions, activated microglia release an inflammasome-dependent formation of apoptosis-associated speck-like protein containing a CARD (ASC) speck, which enhances the formation of Aβ oligomers and aggregates via Aβ binding [38, 42]. These findings support the notion that ASC specks released by microglia following inflammasome activation act as an inflammation-driven cross-seed for Aβ pathology. Thus, we propose that inflammasome activation may play a part in microglial autophagy indirectly affecting Aβ propagation. However, the exact mechanism through which microglial autophagy affects Aβ propagation remains unknown. It would be of interest to determine whether defective microglial autophagy can lead to the secretion of non-degraded Aβ, as in the mechanism of Tau secretion.

Indeed, microglial autophagy is implicated in Tau aggregation and spread. Bolos *et al*. [43] reported that microglia colocalize with various forms of Tau in postmortem brain tissue from AD patients. Further, microglia take up Tau both *in vitro* and *in vivo*. Microglia were also shown to participate in the internalization and degradation of pathological p-Tau isolated from AD brain tissue [44]. In addition, Asai *et al*. demonstrated that microglia spread Tau via exosome secretion and the inhibition of exosome synthesis (while microglia depletion impeded Tau propagation) [45]. Microglia play a complex role in Tau uptake, with inefficient Tau degradation potentially leading to the release of Tau "seeds," a form of Tau that can accumulate in recipient cells [46]. Microglia were reported to become hypophagocytic after phagocytosing Tau aggregate-bearing neurons, whereafter they release Tau seeds, thus acting as vectors of Tau aggregate spreading [47]. These data suggest a direct role of microglia in Tau propagation via seed-competent Tau spreading.

Deletion of microglial *Atg7* in PS19 mice led to elevated p-Tau levels and NFT pathology, which was associated with accelerated Tau seed-mediated spread. Furthermore, autophagy deficiency in microglia led to inflammasome activation and cytokine production, enhancing intraneuronal Tau pathology and its spread [12]. Based on these findings, we propose that microglial autophagy regulates either intraneuronal or extracellular Tau spread via multiple mechanisms. First, defective microglial autophagy causes inflammasome activation and proinflammatory cytokine production, which have been proven to induce Tau phosphorylation in neurons [48-50]. Second, defective autophagy deficiency and the associated heightened microglial activation compromise phagocytosis, impairing extracellular Tau uptake and clearance [51]. Third, defective microglial autophagy may lead to Tau aggregation, thus accelerating microglial dysfunction and resulting in Tau secretion.

Previous studies have proposed the existence of crosstalk between Aβ, Tau pathology, and microglial autophagy [52, 53]. Aβ plaques promote tau aggregation in dystrophic neurites. Meanwhile, injection with protopathic Tau seeds exacerbates Aβ deposits and decreases the number of microglia around Aβ plaques. Improvement of microglial autophagy and plaque-associated microgliosis was shown to promote Tau phagocytosis and reduce Aβ load in APP/PS1 mice [53]. In addition, the imbalance of microglial autophagy in response to the burden of Aβ and p-Tau leads to sustained microglial activation or excessive microglial autophagy, which may result in autophagic death. These results highlight the complex interactions between microglial autophagy, Aβ, and p-Tau clearance in AD pathology. Besides, more research is still needed to establish the role of microglial autophagy in the propagation of Aβ and Tau. Targeting microglial autophagy might provide an opportunity to interfere with the accumulation of these pathological proteins.

### Synaptic dysfunction

Synaptic dysfunction causes cognitive impairment in AD patients. Microglia participate in the formation and maturation of synapses in the CNS [54]. More specifically, microglia play a significant role in synaptic pruning, which is essential for postnatal brain development in mice. Further, deficits in microglial function may contribute to synaptic abnormalities in light of the importance of microglial surveillance during synaptic maturation [54]. Schafer *et al*. further investigated the precise function of microglia in synapse remodeling and revealed the mechanisms underlying microglia-synapse interactions. They found that complement receptor 3(CR3)/C3 expressed on the microglial surface mediates a novel mechanism through which microglia engulf and remodel developing synapses [55]. In mouse AD models, injection with oligomeric Aβ (oAβ) induces an aberrant increase in C1q complement-dependent pathway activity, leading to the phagocytosis of synapses by microglia. Furthermore, blocking Aβ production or the inhibition of C1q deposition ameliorate the oAβ-mediated microglial synaptic engulfment and synapse loss, implying the essential roles for complement and microglia in mediating synapse loss in AD [56].

Evidence supporting the implication of microglial autophagy in synaptic function is available, as abnormal accumulation of autophagic vesicles correlates with axonal and synaptic pathology in the hippocampus of young AD mice [57]. Furthermore, brain-derived neurotrophic factor (BDNF) secreted by microglia increases neuronal tropomyosin-related kinase B phosphorylation and thereby acts as a potent regulator of synaptic development and plasticity [58, 59]. However, microglia-specific autophagy-deficient mice exhibit a reduction of BDNF expression in the brain [60]. Thus, it is unclear whether microglial autophagy can regulate synaptic plasticity through BDNF. In addition, hippocampal accumulation of Aβ induces defective autophagy and mitophagy, causing synaptic damage in hippocampal neurons from AD [61]. Notably, defective microglial autophagy contributes to dysfunctional synaptic pruning and neurobehavior regulation in an autism spectrum disorder mouse model. Besides, impaired synapse degradation and increased numbers of immature synapses have been observed in *Atg7*-deficient microglia *in vitro* [62]. In this regard, we propose a potential link between microglial autophagy and synaptic function in AD, which requires further study.

## AD-related microglial receptors and autophagy

**Figure 2.**
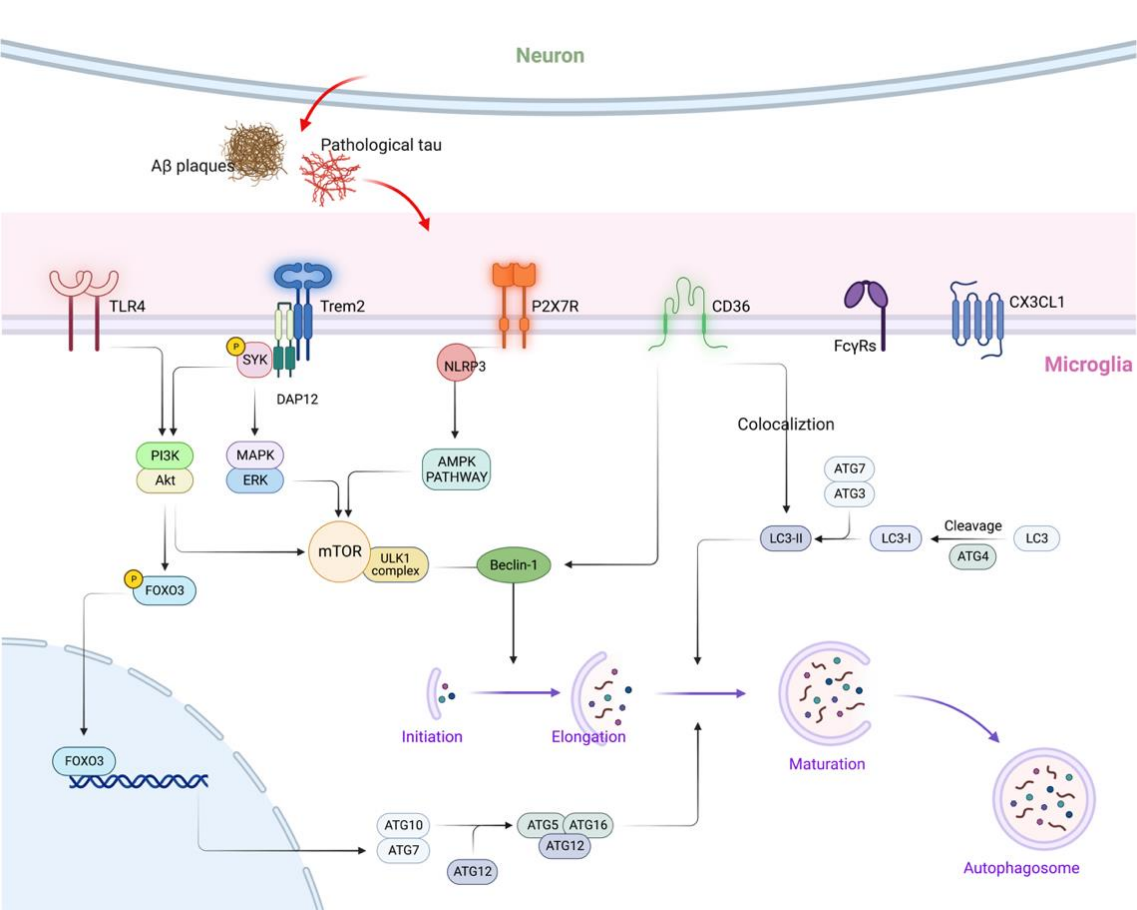
**A model for autophagy mediated by microglial receptors**. Autophagy is influenced by several AD-related microglia receptors, particularly TLR4, TREM2, P2X7R, and CD36. TLR4 activation reduces autophagic flux and the expression of Atg genes in microglia by inhibiting the PI3K-Akt and downstream FOXO3 and mTOR pathways. TREM2 binds DAP12, followed by the recruitment of SYK, leading to PI3K and MAPK activation, thereby triggering mTOR signaling. P2X7R regulates microglial autophagy via AMPK-mTOR signaling. CD36 colocalizes with autophagosome membrane protein MAP1LC3/LC3 and influences the function of beclin-1, thus regulating autophagy.

Microglia express several receptors that cooperate in recognition, internalization, and activation. However, the receptors through which microglia autophagy is provoked remain elusive. Specific microglial receptors can regulate autophagy, phagocytosis, or other microglial functions, thereby participating in AD development. Here, we provide a comprehensive view of the relationship between several microglia-expressing receptors and autophagy regulation in AD (Fig. 2).

### TREM2

TREM2 is an immunomodulatory transmembrane receptor that consists of an extracellular V-type Ig domain, transmembrane helix domain, and a short cytosolic tail. The importance of TREM2 is highlighted by the identification of its variants in AD and other neurodegenerative diseases [63, 64]. In brain tissues, TREM2 is expressed as a cell-surface receptor on microglia, responding to Aβ deposition and limiting neuronal degeneration [65]. Previous studies have demonstrated that TREM2 overexpression decreases autophagy via activation of mTOR signaling in RAW264.7 macrophages [66]. However, the potential link between TREM2 and microglial autophagy has scarcely been addressed. Recently, Ulland *et al*. [67] reported microglia carrying TREM2 risk variants (R47H and R62H) in AD patients, and this displayed marked autophagy activation. Microglia from TREM2-deficient mice (*Trem2*^-/-^ 5×FAD) with AD-like pathology contain abundant autophagic-like vesicles. TMEM59 is a ubiquitously expressed type I transmembrane protein that can promote the ratio of LC3B-II/LC3B-I by enhancing autophagic flux or interfering with autolysosome formation [68]. Liu *et al*. [69] recently reported that TREM2 interacts with TMEM59, most likely through their transmembrane domains. Further, TREM2 overexpression promoted TMEM59 protein degradation in microglia, while in *Trem2* knockout microglia, TMEM59 protein and LC3B-II levels were significantly increased, implying that TREM2 may regulate microglial autophagy through its interaction with TMEM59. In addition, several studies have found that TREM2 is implicated in microglial phagocytosis. Kleinberger *et al*. [70] reported that TREM2 mutations impair phagocytosis. Acute TREM2 downregulation enhances microglial phagocytosis in mice, thus slowing amyloid deposition [71]. The involvement of TREM2 in regulating microglial phagocytosis may provide new insight into its potential role in interfering with autophagy.

Better elucidation of the molecular mechanism underlying TREM2 deficiency-associated alteration of microglia autophagy may provide new insights into AD intervention. TREM2 transmits intracellular signals by binding a lysine residue in the transmembrane domain and its transmembrane binding partner, DAP12 [72]. Upon TREM2 ligand binding, DAP12 is phosphorylated, followed by the recruitment of spleen tyrosine kinase, which thereby triggers a cascade of signaling events, including phosphoinositide 3-kinase (PI3K) activation and MAPK activation [16, 73, 74]. As an important downstream target of PI3K, Akt activates the mammalian target of rapamycin complex 1 (mTORC1), which further regulates autophagy via the phosphorylation of Atg13, ULK1 (Ser758), and DAP1 (Ser3/Ser51). mTORC1 activation leads to AMPK phosphorylation at α1Ser347/α2Ser345, and AMPK can regulate autophagy through direct phosphorylation of ULK1 (Ser 317 and Ser 777) [14, 16]. Furthermore, p38α MAPK directly interacts with ULK1. Activation of p38α MAPK affects the ULK1 kinase activity, disrupts its interaction with Atg13 within the autophagy initiation complex, and eventually reduces autophagic flux [15]. We propose that TREM2 may trigger these intracellular events, such as mTORC1, AMPK, and p38α MAPK pathways, and thus regulate microglia autophagy. However, new insight into the TREM2-mediated mechanism and signaling pathways in microglia autophagy will become an important direction of future research.

### Scavenger receptors (SRs)

SRs are a 'superfamily' of membrane-bound receptors that are classified into 10 families, defined as Classes A-J, with essential roles in the innate immune response and inflammation [75]. SRs are pivotal in major aspects of macrophage biology, including differentiation, migration, and phagocytosis [76]. SR-A is expressed on both microglia and astrocytes. SR-A has emerged as a relevant player in AD, since it was found to participate in Aβ uptake and in modulating the inflammatory response of glial cells. SR-A deficiency affects the microglial inflammatory response and phagocytic activity [77]. In macrophages, SR-A activation via SR-A agonist fucoidan inhibits LC3-II formation and the number of autophagosomes under endoplasmic reticulum stress through the regulation of mTOR signaling [78]. Given that SR-A can regulate macrophage autophagy and microglial phagocytic activity of microglia, we speculate that SR-A may also potentially mediate microglial autophagy regulation. Class B family (SR-B) comprises SR-B1, SR-B2/CD36, and SR-B3. A previous study demonstrated the possible involvement of SR-B1 and CD36 in microglial interaction with amyloidogenic fragments of Aβ protein, mediating AD progression [79]. CD36 is a membrane protein enriched in cells of the monocyte-macrophage system. It is a negative regulator of autophagy. Knocking down CD36 expression in HepG2 and Huh7 cells induced autophagy with increased autophagosome formation and autophagic flux [80]. Another study showed that exogenous CD5-like (CD5L) enhanced autophagy in hepatocytes through the CD36 receptor, thereby attenuating hepatic I/R injury [81]. Additionally, CD36-deficient B cells exhibit impaired autophagy initiation, with CD36 regulating autophagy and colocalizing with autophagosome membrane protein MAP1LC3/LC3 [82]. Pure synthetic nitro-oleic acid dose-dependently increased CD36 expression and interacted with CD36 in RAW264.7 macrophages, restoring autophagy flux [83]. Lucin *et al*. [18] reported that beclin-1 acts as a link between autophagy, retromer trafficking, and receptor-mediated phagocytosis in microglia and mouse brains. Recycling of phagocytic receptors CD36 and TREM2 is a novel function of beclin-1, implying that CD36 may be associated with microglial autophagy and phagocytosis. In addition, the CD36-associated Aβ clearance by phagocytosis is apparent in both microglia and astrocytes [84].

### Purinoceptors

Purinoceptors (P1 and P2 receptors) mediate purinergic signaling, thus regulating inflammatory responses, and being associated with neuroinflammation during AD [85, 86]. Microglia express various ionotropic P2X receptors (P2X4R and P2X7R) and metabotropic P2Y receptors (P2Y1R, P2Y2R, P2Y4R, P2Y6R, and P2Y12R) [87, 88]. Clinical and experimental studies have found that P2X7R is closely associated with neuroinflammation in AD as it is overexpressed in the brains of AD patients, in Aβ-induced microglia, and in the hippocampus of Aβ-injected rats [89]. Under Aβ overload conditions, P2X7R mediates activation of the NLRP3 inflammasome and release of multiple proinflammatory cytokines from the IL-1 family via inducing microglia activation [90, 91]. Notably, P2X7R participates in microglial clearance through the regulation of autophagy via AMPK signaling, and this event is also related to P2X7R-mediated inflammatory response [91-93]. Thus, we speculate that P2X7R-mediated microglial autophagy may be a strategy for the prevention of AD development. Moreover, while microglial P2X4R [94], P2Y6R [95], and P2Y12R [96] have been implicated in distinct microglial responses during AD [97], their participation in microglial autophagy remains to be confirmed.

### Toll-like receptors (TLRs)

In the human brain, microglia express various TLRs (TLR1-9) [98], among which TLR2 and TLR4 are considered the major functional subtypes [99]. In the early stages of AD, Aβ can directly interact with TLR2 and TLR4 to induce microglial Aβ phagocytosis. In the late stage, Aβ and damage-associated molecular patterns bind and activate TLR2 and TLR4, thus triggering signaling that leads to the activation of proinflammatory transcription factors NF-κB and AP1, thereby generating an inflammatory feed-forward loop [100]. Furthermore, several studies have demonstrated novel roles for TLR2 and TLR4 in regulating microglial autophagy. Arroyo *et al*. [101, 102] reported that TLR2 stimulation enhances microglial autophagy via PI3K. TLR4 activation by lipopolysaccharide (LPS) reduces autophagic flux and *Atg* gene expression in microglia by inhibiting the PI3K-FOXO3 pathway. In addition, TLR4 activation impairs the phagocytic capacity of microglia [103]. Autophagy-associated TLRs, TLR7, TLR8, and TLR9, can enhance Aβ uptake in microglia during the early stage of AD and then contribute to neuroinflammation in the late stage [104]. However, their roles in regulating microglial autophagy are unknown at present. A better understanding of TLRs in microglial autophagy may be beneficial for increasing autophagy and phagocytosis of Aβ and AD inflammatory response reduction.

### Other types of receptors

In addition to the above-described receptors, several other microglial receptors are essential in regulating autophagy in AD. Peroxisome proliferator-activated receptor γ (PPARγ) is involved in AD by restoring microglial function. The PPARγ antagonist promotes the LPS-induced M1-to-M2 phenotypic shift of microglia, which is attributed to the promotion of microglial autophagy [105]. Luo *et al*. [34] report that PPARα regulates autophagy in human microglia cells, and PPARα-mediated autophagy affects Aβ clearance and cognitive decline in a murine AD model. CX3CL1/fractalkine expressed on neurons binds to its receptor CX3CR1 on microglia, thus regulating microglial function [106]. Emerging data have highlighted the involvement of CX3CL1/CX3CR1 signaling in AD pathogenesis. CX3CR1 deficiency contributes to neuroinflammation by upregulating IL-1β and the subsequent induction of microglial autophagy [107]. However, there is no evidence for the direct impact of CX3CR1 deficiency on microglial autophagy in AD. In addition, several receptors, including the tumor necrosis factor receptor TNF-1R, interleukin receptor IL-1R, Fc gamma receptors, and complement receptors, regulate microglial activation and microglia-mediated neuroinflammation, thus participating in AD pathogenesis [108-110]. The role of these receptors in microglial autophagy regulation should be further explored.

## Conclusion

In recent decades, evidence has shown that defective microglial autophagy affects inflammasome activation, inflammatory cytokine production, clearance and prion-like propagation of Aβ and p-Tau, and synaptic dysfunction as a factor that exacerbates AD. Consequently, microglial autophagy regulation should be considered in AD prevention and treatment. Data demonstrates that some microglia-expressing receptors, such as TREM2, P2Rs, SRs, and TLRs, regulate the autophagic process in microglia and are involved in AD pathogenesis. Microglial autophagy-focused strategies considering these receptors may represent a novel approach for AD treatment.
